# Long-Term Conservation of Ohnologs Through Partial Tetrasomy Following Whole-Genome Duplication in Salmonidae

**DOI:** 10.1534/g3.119.400070

**Published:** 2019-04-22

**Authors:** Matthew A. Campbell, Matthew C. Hale, Garrett J. McKinney, Krista M. Nichols, Devon E. Pearse

**Affiliations:** * Fisheries Ecology Division, Southwest Fisheries Science Center, Santa Cruz, CA 95060; † Department of Biology, Texas Christian University, Fort Worth, TX 76129; ‡ School of Aquatic and Fishery Sciences, University of Washington, Seattle, WA 98195, and; § Conservation Biology Division, Northwest Fisheries Science Center, Seattle, WA 98112

**Keywords:** Salmonidae, Ohnologs, Homeologs, Differential Gene Expression, Smoltification

## Abstract

Whole-genome duplications (WGDs) have occurred repeatedly and broadly throughout the evolutionary history of eukaryotes. However, the effects of WGD on genome function and evolution remain unclear. The salmonid WGD that occurred approximately 88 million years ago presents an excellent opportunity for studying the effects of WGD as ∼10–15% of each salmonid genome still exhibits tetrasomic inheritance. Herein, we utilized the rainbow trout (*Oncorhynchus mykiss*) genome assembly and brain transcriptome data to examine the fate of gene pairs (ohnologs) following the salmonid whole-genome duplication. We find higher sequence identity between ohnologs located within known tetrasomic regions than between ohnologs found in disomic regions, and that tetrasomically inherited ohnologs showed greater similarity in patterns of gene expression and per ohnolog were lower expressed, than disomically inherited ohnologs. Enrichment testing for Gene Ontology terms identified 49 over-represented terms in tetrasomically inherited ohnologs compared to disomic ohnologs. However, why these ohnologs are retained as tetrasomic is difficult to answer. It could be that we have identified salmonid specific “dangerous duplicates”, that is, genes that cannot take on new roles following WGD. Alternatively, there may be adaptive advantages for retaining genes as functional duplicates in tetrasomic regions, as presumably, movement of these genes into disomic regions would affect both their sequence identity and their gene expression patterns.

Whole-genome duplication (WGD) events are widespread in the eukaryotic tree of life (*e.g.*, Campbell *et al.*, 2016; Soltis and Soltis 1999; Van de Peer *et al.*, 2017). WGDs create copies of the same gene – termed ohnologs – and this genetic redundancy has long been thought to provide the molecular blueprint for evolution and adaptation (*e.g.*, Ohno 1970). While the influence of WGDs on long-term processes like organismal diversification are unclear (*e.g.*, Mayrose *et al.*, 2011; Santini *et al.*, 2009; Wood *et al.*, 2009; Zhan *et al.*, 2014), evidence suggests that WGDs present a variety of challenges during both mitosis and meiosis (Hollister 2015). These challenges are reflected by the observation that most neopolyploid species revert to diploidy soon after duplication (Gerstein and Otto 2009; Wolfe 2001). Following WGD, ohnologs typically follow four basic patterns: (1) Nonfunctionalization where through mutation the new duplicate loses functionality (Langham *et al.*, 2004), (2) Subfunctionalization in which the duplicates retain subsets of the original gene function (Force *et al.*, 1999; Sémon and Wolfe 2008), (3) Neofunctionalization in which a gene copy takes on a new function (Muller 1936; Ohno 1970; Rastogi and Liberles 2005), and, (4) Conservation of the same function by both copies of the gene (Dean *et al.*, 2008; Tischler *et al.*, 2006; Vavouri *et al.*, 2008). Understanding why some genes are able to become neo- or sub-functionalized while others are lost or conserved is critical for understanding the reversion to diploidy characteristic of polyploid genomes.

Previous research has indicated that the most common fate of a duplicated gene is to become nonfunctionalized and lost (Lynch and Conery 2000). For example, various estimates across fishes suggest that only 12–24% of all genes have been retained as functional duplicates following the teleost WGD 350 million years ago (*e.g.*, Braasch and Postlethwait 2012; Kassahn *et al.*, 2009; Postlethwait *et al.*, 2000; Woods *et al.*, 2005). However, the retention of some ohnologs following duplication indicates that these ohnolog pairs must have experienced functional divergence either by subfunctionalization or neofunctionalization (Braasch *et al.*, 2018, 2016; Sandve *et al.*, 2018). Subfunctionalization leads to limited changes to gene function, as both genes within an ohnolog pair contribute to the original function either by maintaining gene expression to pre-duplication levels, or by obtaining mutations that do not change the functions of either protein (Force *et al.*, 1999; Stoltzfus 1999). Neofunctionalization, on the other hand, results in two genes with different functions – the original function is conserved by one gene copy while mutation of the second gene copy leads to the development of a new function. Neofunctionalization and subfunctionalization are not necessarily mutually exclusive processes and subfunctionalization may be an intermediate step toward neofunctionalization (Rastogi and Liberles 2005).

Lastly, ohnologs may be conserved as redundant pairs with both copies retaining the original functions. Selection for redundancy may be due to different processes depending on the functions of the ohnologs. For example, ohnologs connected to haploinsufficent phenotypes, ohnologs with an increased likelihood of acquiring dominant deleterious mutations (the “dangerous duplicates” hypothesis; *e.g.*, Singh *et al.*, 2012), or when the expression of each ohnolog is required for regular function (the dosage-compensation model (Edger and Pires 2009; Hughes *et al.*, 2007; Pires and Conant 2016) may all explain why some ohnologs are conserved. One way to differentiate between these hypotheses is to investigate the function of retained duplicates. For example Singh *et al.* (2015) proposed that mutations in ohnologs are three times as likely as mutations in non-ohnologs to be associated with autosomal dominant diseases and up to eight times more likely to encode for genes with autoinhibitory protein folds (Singh *et al.*, 2012; 2015; *i.e.*, the “dangerous duplicates hypothesis”). On the other hand, genes involved in processes such as metabolism, transcription, and translation appear to exhbit a reduction in expression in both copies compared to non-duplicates (*i.e.*, the “dosage-balance hypothesis”: Aury *et al.*, 2006; Hughes *et al.*, 2007). In order to more fully understand how patterns of gene expression link with retaining ohnologs post WGD, more data from a wider range of taxa are required.

The common ancestor of salmonid fishes has been identified as an autopolyploid that experienced a whole-genome duplication approximately 88 million years ago (Allendorf and Thorgaard 1984; Berthelot *et al.*, 2014; Macqueen and Johnston 2014). Since then, salmonid genomes have mostly reverted to diploidy (Lien *et al.* 2016), with lineage-specific divergences in ohnolog function (Robertson *et al.* 2017). This re-diploidization is demonstrated by the reduction in the number of protein coding genes from a theorized 62,952 in the ancestral tetraploid salmonid to 46,585 in the modern rainbow trout genome (Berthelot *et al.*, 2014). However, there is evidence that 10–15% of most salmonid genomes are inherited tetrasomically, with eight chromosome arm pairs commonly forming tetravalents during meiosis (Allendorf *et al.* 2015). Strikingly, the homologs of these eight chromosome arm pairs have independently maintained tetrasomy during the evolution of *Salmo*, *Salvelinus*, and *Oncorhynchus* despite multiple fusion and fission events between chromosome arms across species (*e.g.*, Brieuc *et al.*, 2014; Kodama *et al.*, 2014; Lien *et al.*, 2011; Sutherland *et al.*, 2016; Waples *et al.* 2016).

Phylogenetic correlation could potentially explain the retained duplication patterns for the eight homeologous arms at recent time scales; however, the conservation of tetrasomic inheritance by the same genes and genomic regions across *Salmo*, *Oncorhynchus*, and *Salvelinus* despite tens of millions of years of independent divergence among these lineages strongly supports underlying selection *vs.* phylogenetic correlation. The conservation of these eight chromosome arm pairs is well known, yet, there is limited information regarding why these regions are maintained as tetrasomic or how their tetrasomic inheritance influences patterns of gene expression.

The aims of this manuscript are threefold: (1) to compare sequence similarity between tetrasomically and disomically inherited ohnologs; (2) to compare and contrast patterns of gene expression between tetrasomically and disomically inherited ohnologs; and (3) to compare and contrast functionality of ohnologs – as inferred from Gene Ontology terms - between tetrasomic and disomically inherited regions of the genome. To accomplish these aims we use the newly updated *Oncorhynchus mykiss* (rainbow and steelhead trout) genome assembly (https://www.ncbi.nlm.nih.gov/assembly/GCF_002163495.1/; Pearse *et al.*, 2018) and gene expression data from a population of rainbow trout that has become a model system for understanding the genetic basis of migration (Hale *et al.*, 2016; 2013; Hecht *et al.*, 2015; 2012; McKinney *et al.*, 2015). Here we demonstrate that ohnolog pairs in tetrasomic regions are more similar in their sequence identity than those in disomic regions of inheritance, and that patterns of gene expression are more similar between ohnologs in tetrasomically inherited regions than between ohnologs in disomic regions. GO enrichment identified 49 over-represented terms among ohnologs in tetrasomically inherited genomic regions, indicative of differences in function between inheritance modes. These results provide insight into the underlying constraints that sustain tetrasomic inheritance in these regions of the genome.

## Methods

### Ohnolog identification, similarity and inheritance mode

A table of ohnolog pairs arising from the salmonid-specific autotetraploidization event (Ss4R, Berthelot *et al.* 2014) that includes overall similarity between each member of a pair (percent identity in terms of DNA similarity, PID) and genomic locations of both members of a pair was generated following methods used in Lien *et al.* (2016). Briefly, an all-*vs.*-all BLASTP (Altschul *et al.*, 1990) of amino acid translations of the longest transcript for each locus was performed with the following guidelines: minimum alignment length of 50% and minimum percentage sequence identity of 85%. The top ranked BLASTP hits from known Ss4R homeolog regions (see Pearse *et al.* 2018) were identified. Ohnologs were then divided into six categories based on overall DNA sequence similarity between both members of the pair, (<90%, 90–95%, and >95% PID, similar to other salmonid studies, *e.g.*, Lien *et al.* 2016), and inheritance mode (tetrasomic or disomic) as determined by the location of the ohnologs in the rainbow trout genome. It is well known that homeologous regions of the salmonid genome form tetravalent meioses in *Oncorhynchus*, *Salmo*, and *Salvelinus* (Kodama *et al.*, 2014; Lien *et al.*, 2016; Sutherland *et al.*, 2016); therefore, positional information on the rainbow trout genome was deemed as sufficient evidence for tetrasomic inheritance.

To visualize the relationship between ohnolog PID and inheritance mode, we plotted the chromosome position on the *x* - axis and PID of each ohnolog to its partner on the *y* - axis while splitting by inheritance mode (*i.e.*, tetrasomic or disomic). The PID values were plotted using a smoothed conditional means approach (geom_smooth function of ggplot) to avoid over plotting and increase visibility of patterns. Overall differences in ohnolog sequence similarity were characterized by generating histograms of percent similarity of ohnolog pairs within disomic and tetrasomic regions of the rainbow trout genome, with each histogram scaled to integrate to 1.0 for comparative purposes (geom_histogram function in R package ggplot2). We tested the difference in mean PID of ohnolog pairs between disomic and tetrasomic genome regions with a Welch Two Sample t-Test (t.test function of base R with alpha = 0.05). All analysis and figures were constructed using R version 3.3.1 (R Development Core Team 2017) and the ggplot2.2.1 package (Wickham 2009).

### Gene expression analysis

To compare patterns of gene expression between ohnolog pairs, RNA-seq data were used from a previous study examining differences in gene expression between migratory and non-migratory *O. mykiss* from Sashin Creek, Alaska (NCBI Sequence Read Archive under the BioProject Number PRJNA269115; Hale *et al.*, (2016)). Briefly, two-year-old fish originating from two type of crosses - one between two migratory anadromous parents (A x A) and one between two resident parents (R x R) - were reared in a common garden experiment at the Little Port Walter Marine Station to examine developmental gene expression using RNA-seq (Hale *et al.*, 2016). At age 2, seven fish from the A x A line (n = 4 females and n = 3 males) and eight fish from the R x R line (n = 4 females and n = 4 males) were killed using a lethal dose of buffered MS222. Whole brains were dissected and stored immediately in RNAlater (ThermoFisher). Complete details of RNA extraction, library preparation, and sequencing are described in Hale *et al.* (2016).

The study herein constitutes a re-analysis of the data presented in Hale *et al.* (2016). Instead of a *de novo* assembly, the most recent version of the rainbow trout genome was used as a reference to map RNA-seq reads (NCBI accession number: GCF_002163495.1; Pearse *et al.*, 2018). Prior to alignment, RNA-seq reads were filtered for low quality bases (Phred scores < 33) and reads less than 25 base pairs (bp) were discarded with Trimmomatic version 0.36 (Bolger *et al.*, 2014). Cleaned reads were mapped to assembled chromosomes of the rainbow trout genome with hisat2 version 2.0.4 under the default settings (Kim *et al.*, 2015). The resulting Sequence Alignment Map (SAM) files were processed by converting to sorted binary files (BAM) using SAMtools version 1.3 (Li *et al.*, 2009). From BAM files, the htseq-count script of HTSeq version 0.6.1 (Anders *et al.*, 2015) was used to generate read count matrices with the option “-m union” specified to allow reads that align over part of a gene or across exons to be counted while considering those reads aligning over parts of two genes to be ambiguous.

To examine global expression differences between ohnolog pairs located in disomic and tetrasomic genomic regions we examined the total expression matrix and divided it by cross type. We further divided the expression data matrix by each cross type and sex (four total data sets: A x A Females, A x A Males, R x R Females, R x R Males). For each of these cross type and sex data sets (7 total) expression between ohnolog pairs was calculated as the absolute value of log_2_ fold change with DESeq2 (Love *et al.*, 2014), providing a measure of expression difference between copies of an ohnolog pair specific to each cross and sex. We visualized resulting log_2_ fold change measures with two-dimensional kernel density estimates of the absolute value of log fold change between the two genes in an ohnolog pair split by inheritance mode for each of the four data sets resulting from splitting by cross type and sex (stat_density_2d function of ggplot with R version 3.3.1) (R Development Core Team 2017; Wickham 2009). For the *x* - axis, we generated relative positions of the ohnolog by dividing the position of the ohnolog by the total length of the chromosome. The *y* - axis component is an expression of absolute fold change, presented as the absolute value of log_2_ fold changes found between ohnologs calculated by DESeq2, to show differences without direction (negative or positive fold changes). We examined the mean and variance of the absolute value of log_2_ fold changes between ohnolog pairs of different inheritance modes (summary function of base R) and tested for differences between the means with a Welch t-Test (t.test function of base R).

To identify genes and ohnologs that may have sex-specific or life history-specific function, we tested for differential expression between cross types, A x A (n = 7) *vs.* R x R (n = 8), and between cross types split by sex. That is, read count data were further subdivided by both cross type and sex, and were analyzed as A x A females (n = 4) *vs.* R x R females (n = 4) or A x A males (n = 3) *vs.* R x R males (n = 4). To be tested for differential expression, a gene was required to have at least five reads mapping to it when summed across individuals. Differential expression was tested by DESeq2 with the “DESeq” function which includes normalization of raw read counts as described in Love *et al.*, (2014). We tested all anadromous individuals against all resident individuals with sex considered a cofactor within the model. For sex-specific models, this co-factor was not included. Significance was designated with an adjusted *p* – value < 0.1 in DESeq2 with an implementation of the Benjamini-Hochberg false discovery rate method (Benjamini and Hochberg 1995).

### Gene ontology (GO) analysis

Ohnologs were classified as being inherited via disomy or tetrasomy based on their location in the rainbow trout genome (Pearse *et al.*, 2018). All 7,879 ohnolog pairs were annotated by using BLAST against the UniProt database (downloaded 10-12-2017) using a maximum e-value of 1.0 × 10^−8^ and default parameters. Associated Gene Ontology (GO) terms were mapped using Blast2GO with default parameters. Fisher’s exact tests were used for testing enrichment of GO terms in all tetrasomic ohnologs compared to all disomic ohnologs. A maximum *p* – value cut off of 0.001 was used to infer enrichment.

### Protein complexes

In order to identify differences in protein-protein interactions between ohnologs located in disomic regions and ohnologs located in tetrasomic regions, we obtained the number of subunits each protein encoded by ohnologs interacted with. These data were manually downloaded from the UniProtKB/Swiss-Prot database on January 26, 2019. Information on protein interactions was downloaded for three organisms: Atlantic salmon, zebrafish and mouse. A Perl script (extract_complex_info_UniProt.pl, available at https://github.com/julien-roux/Roux_Liu_and_Robinson-Rechavi_2016) designed by Roux *et al.* (2017) was used to identify the “subunit structure” annotation category linked to the annotated ohnologs. The following complex categories were utilized by Roux *et al.* (2017) and by this study: monomers, homomultimers, heterodimers, heteromultimers (more than two different subunits), and “other complexes” for descriptions that could not be automatically categorized. We tested if the proportions of complex categories from annotated ohnologs differed between disomic and tetrasomic ohnologs with a *X*-squared test (prop.test function in base R).

### Data availability

A table of rainbow trout ohnologs originating from the Ss4R as described is provided as Supplemental File S1. Supplemental File S2 contains summary alignment statistics from the HTSeq analysis. Supplemental File S3 contains information on gene expression from DeSeq2 including log_2_ fold difference in expression, raw, and FDR corrected *p* –values. Supplemental File S4 shows the specific genes associated with enriched GO terms in tetrasomic regions of the rainbow trout genome. Supplemental File S5 shows differentially expressed genes between cross type with associated GO terms. Supplemental File S6 shows proportions of disomic and tetrasomic ohnologs with protein-protein interaction information. All RNA-seq data used in this manuscript have been uploaded to NCBI (BioProject Number PRJNA269115) and the rainbow trout genome can be accessed at https://www.ncbi.nlm.nih.gov/assembly/GCF_002163495.1/. Supplemental material available at FigShare: https://doi.org/10.25387/g3.7732358.

## Results

### Ohnolog identification, similarity and inheritance mode

Of the 7,979 ohnolog pairs originating from the Ss4R identified from the rainbow trout genome, 5,701 (72.4%) were predicted to be inherited disomically and 2,278 (27.6%) were predicted to be inherited tetrasomically based on their locations on the rainbow trout genome (Table 1, Supplemental File S1). Most disomically inherited ohnologs showed low (*i.e.*, less than <90%) sequence similarity, whereas most tetrasomically inherited ohnologs show very high (>95%) sequence similarity, and ohnolog pairs within tetrasomic regions showed greater similarity (PID) than those located in disomic regions (Figures 2 & 3). This difference was statistically significant as mean disomic PID per ohnolog = 91.28, n = 11,402, was lower than the mean tetrasomic PID per ohnolog = 94.98, n = 4,556 (t = -43.989, df = 9133.3, p-value < 2.2e-16). Despite the high sequence similarity for ohnologs in most tetrasomic pairings, ohnologs on Omy01q and Omy23 had lower levels of sequence similarity (only 25% of ohnologs had >95% PID) whereas most ohnolog pairs in these regions had medium (90–95%) or low (<90%) sequence similarity (Figure 4). This result confirms previous studies in Atlantic salmon (Lien *et al.*, 2016) and rainbow trout (Pearse *et al.* 2018) that found that the homologs of Omy01q and Omy23 (Ssa18qa and Ssa01qa) show reduced evidence of tetrasomic inheritance compared to the other seven tetrasomic pairings.

**Table 1:** Summary of ohnologs split by identity and location in genome. Ohnolog pairs are divided into three categories of overall similarity in terms of DNA sequence similarity for both tetrasomic and disomic pairs, and the percentage of each category is presented

Similarity Category	Tetrasomic Ohnolog Pairs	Category as Percentage	Disomic Ohnolog Pairs	Category as Percentage
>95%	1,410	62%	1,591	28%
95–90%	528	23%	1,950	34%
<90%	340	15%	2,160	38%
Total	2,278		5,701	

### Gene expression analysis

On average, the fifteen rainbow trout samples from PRJNA269115 had 18,420,094.13 reads aligned to genes in the rainbow trout genome (Minimum 4,404,216, Maximum 25,608,943, Supplemental File S2). In the four sample groups (A x A Female, A x A Male, R x R Female, and R x R Male) the vast majority of ohnolog pairs were expressed (from 7,802 to 7,860) in all samples (Table 2). The absolute values of log_2_ fold changes in expressed genes were consistently lower between ohnologs located in tetrasomic regions than between pairs in disomic regions in all seven comparisons of the expression data (t = 8.8826 to 9.6825, *P* < 0.001 in all contrasts; Table 2). Visualizations of expression data shows that tetrasomic genes cluster toward the ends of chromosomes and exhibit less ohnolog to ohnolog variation in expression than those in disomic regions (Figure 5).

**Table 2:** For each cross split by sex the mean and variance for the absolute value of log_2_ fold change for both disomically and tetrasomically inherited ohnologs is presented. The results of Welch t-Tests testing for a difference in means with the 95% confidence interval of the test are presented

Cross Type	Sex	Mean Absolute Value of Log_2_ Fold Change between ohnologs		Variance Absolute Value of Log_2_ Fold Change between ohnologs		Welch t-Test Results	0.95
		Disomic	Tetrasomic	Disomic	Tetrasomic		
A x A	F	2.11	1.78	5.22	4.14	t = 8.8826, df = 9147.3, p-value < 2.2e-16	0.26 - 0.40
A x A	M	2.11	1.76	5.02	3.94	t = 9.4028, df = 9107.8, p-value < 2.2e-16	0.28 - 0.42
A x A	All	2.08	1.74	5.04	3.98	t = 9.2441, df = 9178.2, p-value < 2.2e-16	0.27 - 0.41
R x R	F	2.08	1.72	5.38	4.21	t = 9.5302, df = 9186.5, p-value < 2.2e-16	0.29 - 0.43
R x R	M	2.08	1.73	5.36	4.16	t = 9.2808, df = 9197.5, p-value < 2.2e-16	0.28 - 0.42
R x R	All	2.05	1.69	5.24	4.03	t = 9.6825, df = 9294.2, p-value < 2.2e-16	0.29 - 0.43
Both	All	2.04	1.69	5.08	3.96	t = 9.5941, df = 9255.6, p-value < 2.2e-16	0.28 - 0.42

Of the 42,087 expressed genes in the brain transcriptome, 367 are upregulated in A x A fish and 209 in R x R fish when testing for differential expression with sex as a co-factor (n = 8 and n = 7, Supplemental File S3). When cross types were further divided by sex, 212 genes were differentially expressed between cross types in at least one contrast, of which, 26 genes were upregulated in A x A females, 30 were upregulated in R x R females, 40 genes were upregulated in A x A males, and 116 were upregulated in R x R males. Of these differentially expressed genes, eight and seven were ohnologs in the A x A females and R x R females respectively, and 17 and 41 were ohnologs in the R x R females and R x R males respectively, indicating that some genes in the brain transcriptome and potentially important in the development of anadromy are ohnologs.

### GO analysis

A total of 7,692 ohnolog pairs (out of 7,979) were annotated against the UniProt database. Enrichment analysis revealed that 49 GO terms were overrepresented in tetrasomically inherited ohnologs compared to disomic ohnologs from 185 genes (Table 3; Supplemental File S4). These terms represent an enrichment of functions connected to iron binding and respiration, with three Molecular Functions— iron ion binding, oxidoreductase activity, and oxygen binding— enriched in both cross types. Genes annotated with these GO terms included many myosin, cytochrome P450, and hemoglobin genes, suggesting conservation of genes connected to respiration and gaseous exchange being maintained in tetrasomic regions of high sequence similarity (see Supplemental File S4 for a full list of genes annotated with enriched GO terms).

**Table 3:** Enriched GO terms in tetrasomic high similarity (>95% protein similarity). GO categories are abbreviated to BP (biological process), MF (molecular function), and CC (cellular component. P-values following a Fisher’s exact test are reported

GO ID	GO Term		P-value
GO:0051295	establishment of meiotic spindle localization	BP	4.38E-05
GO:0010728	regulation of hydrogen peroxide biosynthetic process	BP	9.27E-05
GO:0010248	establishment or maintenance of transmembrane electrochemical gradient	BP	1.29E-04
GO:0050665	hydrogen peroxide biosynthetic process	BP	3.80E-04
GO:0090662	ATP hydrolysis coupled transmembrane transport	BP	6.41E-04
GO:1903919	negative regulation of actin filament severing	BP	9.22E-04
GO:0010729	positive regulation of hydrogen peroxide biosynthetic process	BP	9.22E-04
GO:0032796	uropod organization	BP	1.10E-03
GO:1903918	regulation of actin filament severing	BP	1.10E-03
GO:0021678	third ventricle development	BP	1.13E-03
GO:0040023	establishment of nucleus localization	BP	1.22E-03
GO:0006958	complement activation, classical pathway	BP	1.89E-03
GO:0070602	regulation of centromeric sister chromatid cohesion	BP	1.89E-03
GO:0007097	nuclear migration	BP	2.07E-03
GO:0001778	plasma membrane repair	BP	2.41E-03
GO:0030241	skeletal muscle myosin thick filament assembly	BP	2.75E-03
GO:0071688	striated muscle myosin thick filament assembly	BP	2.75E-03
GO:0002455	humoral immune response mediated by circulating immunoglobulin	BP	2.85E-03
GO:0010726	positive regulation of hydrogen peroxide metabolic process	BP	3.02E-03
GO:0021592	fourth ventricle development	BP	3.02E-03
GO:0060004	reflex	BP	5.58E-03
GO:0031034	myosin filament assembly	BP	5.75E-03
GO:0030220	platelet formation	BP	6.29E-03
GO:0036344	platelet morphogenesis	BP	6.29E-03
GO:0070162	adiponectin secretion	BP	6.63E-03
GO:0070163	regulation of adiponectin secretion	BP	6.63E-03
GO:1903921	regulation of protein processing in phagocytic vesicle	BP	6.80E-03
GO:1903923	positive regulation of protein processing in phagocytic vesicle	BP	6.80E-03
GO:1900756	protein processing in phagocytic vesicle	BP	6.80E-03
GO:0048172	regulation of short-term neuronal synaptic plasticity	BP	7.58E-03
GO:1904707	positive regulation of vascular smooth muscle cell proliferation	BP	8.64E-03
GO:0060283	negative regulation of oocyte development	BP	8.64E-03
GO:0070989	oxidative demethylation	BP	8.65E-03
GO:0031449	regulation of slow-twitch skeletal muscle fiber contraction	BP	8.65E-03
GO:0031444	slow-twitch skeletal muscle fiber contraction	BP	8.65E-03
GO:0008635	activation of cysteine-type endopeptidase activity involved in apoptotic process cytochrome c	BP	8.65E-03
GO:0005826	actomyosin contractile ring	CC	1.54E-04
GO:0005833	hemoglobin complex	CC	2.85E-03
GO:0016705	oxidoreductase activity, acting on paired donors, with incorporation or reduction of oxygen	MF	5.14E-05
GO:0005506	iron ion binding	MF	1.95E-04
GO:0019825	oxygen binding	MF	3.16E-04
GO:0043495	protein anchor	MF	2.75E-03
GO:0030250	guanylate cyclase activator activity	MF	6.63E-03
GO:0030249	guanylate cyclase regulator activity	MF	6.63E-03
GO:0019785	ISG15-specific protease activity	MF	6.63E-03
GO:0010853	cyclase activator activity	MF	6.63E-03
GO:0005344	oxygen transporter activity	MF	6.80E-03
GO:0004030	aldehyde dehydrogenase [NAD(P)+] activity	MF	8.65E-03
GO:0016840	carbon-nitrogen lyase activity	MF	8.65E-03

Differentially expressed genes between cross types were generally found in disomic rather than tetrasomic regions of the genome. Most of the differentially expressed genes in disomic regions were associated with GO terms connected to functions such as nervous system development, neuron differentiation, brain development and gland development (Supplemental File S5). Largely the same under-represented GO terms were assigned to genes upregulated in R x R fish. Only one gene that was differentially expressed had a GO term that is over-represented in tetrasomic genomic regions. The gene CIGENEomyV6.45980 located on Omy13q, with the GO term “signal transducer activity” was upregulated in the A x A fish compared to the R x R (Supplemental File S5).

### Protein complexes

We obtained 11,135 disomic ohnologs and 4,336 tetrasomic ohnologs from the rainbow trout genome annotations that also contained UniProtKB accessions. From testing for different proportions of protein-protein interactions between disomic and tetrasomic ohnologs, only two significantly different proportions were detected. The mouse UniProt data indicated that the proportion of tetrasomic ohnologs identified as monomers is ∼1.7 times that of disomic ohnologs and significantly different (*p* – value = 0.019). Additionally, the proportion of tetrasomic ohnologs identified as heterodimers is ∼1.4 times that of disomic ohnologs and significantly different (*p* – value = 0.032). The full results for all three references are presented in Supplemental File S6.

## Discussion

**Figure 1:**
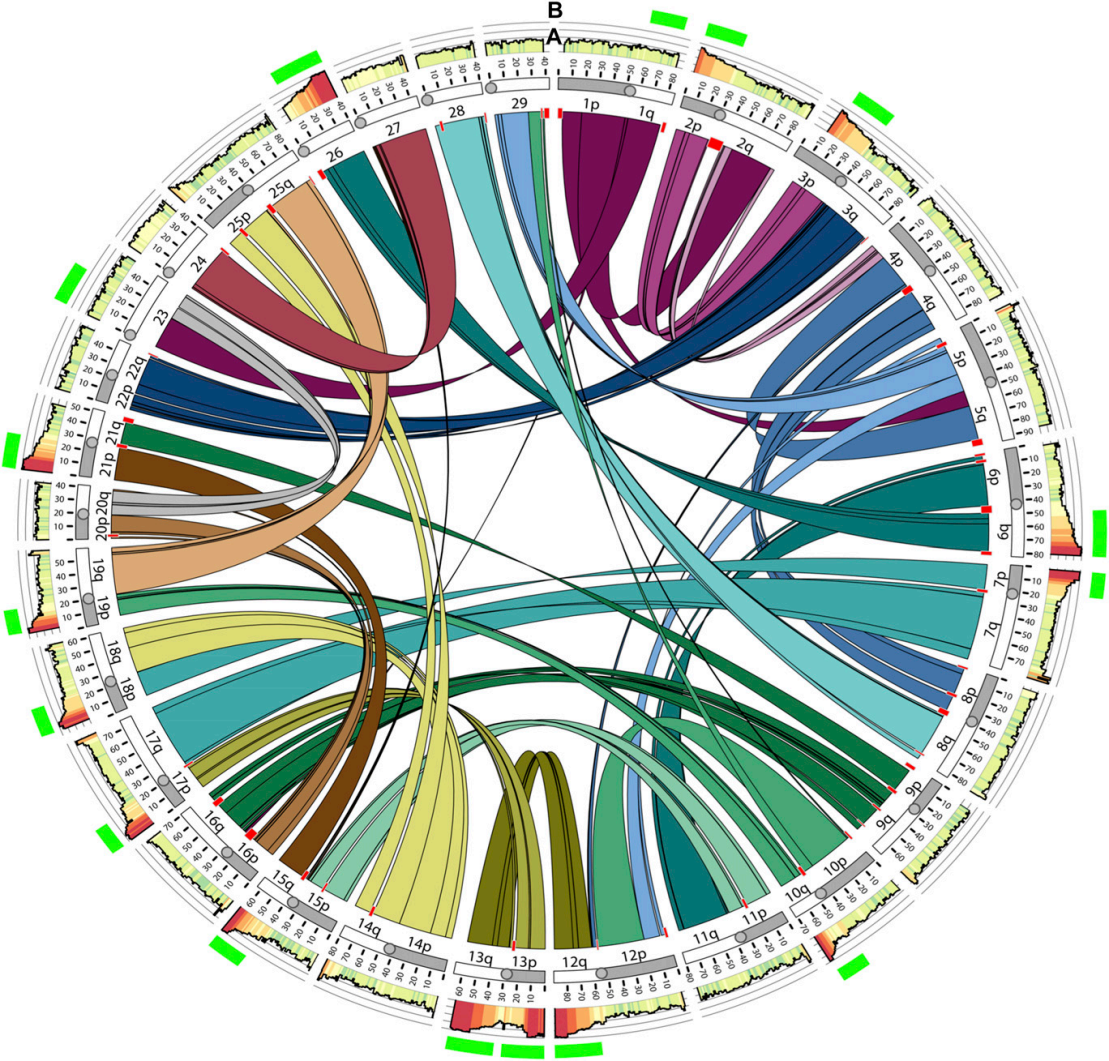
Circos plot showing homeologous blocks in the rainbow trout genome dating from the last whole-genome duplication in salmonids. Chromosomes (Omy01-Omy29) are plotted, arms (*e.g.*, 1p, and 1q) are shaded, and the length of the chromosomes are indicated. Links show pairing between genomic regions originating from duplication at the Ss4R. Track A, overall similarity between homeologous regions is indicated by shading, yellow - low, red - high, and height of the plot. Track B. The eight pairs of homeologous blocks still exhibiting tetrasomic inheritance are indicated by green bars in the outer ring. Circos plot adapted from Pearse *et al.* (2018).

All vertebrates are descended from a common ancestor with two historic WGD events (Blomme *et al.*, 2006; Dehal and Boore 2005; Nakatani *et al.*, 2007; Vandepoele *et al.*, 2004). However, few subsequent vertebrate WGDs have been documented, which makes investigating the fate of ohnologs difficult, as the genomes of most vertebrates have reverted to diploidy (Mable 2004; Orr 1990). To that end, the teleosts are an excellent group for studying the effects of WGD as not only do they share a third WGD event (∼350 million years ago), but several lineages also have independently undergone a fourth WGD event (*e.g.*, the catostomids, cyprinids, and salmonids: Allendorf and Thorgaard 1984; Ohno *et al.*, 1967; Uyeno and Smith 1972). These more recent WGD events provide opportunities to ask questions about how WGD affects genome organization and how the rate of diploidization varies between species and among different regions of the genome. Although gene duplication has long been hypothesized to generate novelty by permitting adaptation via the evolution of new molecular functions (*i.e.*, neofunctionalization; Ohno 1970), here we demonstrate that gene conservation is also a substantial outcome of WGD, at least for salmonids. For example, despite the fact that only 10% of the rainbow trout genome is tetrasomically inherited, ∼28% of conserved ohnolog pairs are located in tetrasomic regions, indicating that tetrasomic inheritance suppresses the process of neofunctionalization and molecular adaptation (see Figure 1). In addition, we demonstrate that tetrasomic ohnologs are conserved in both function and expression and are enriched for molecular processes that might play important roles in salmonid life history adaptation.

### Conservation of the ‘Magic Eight’

**Figure 2:**
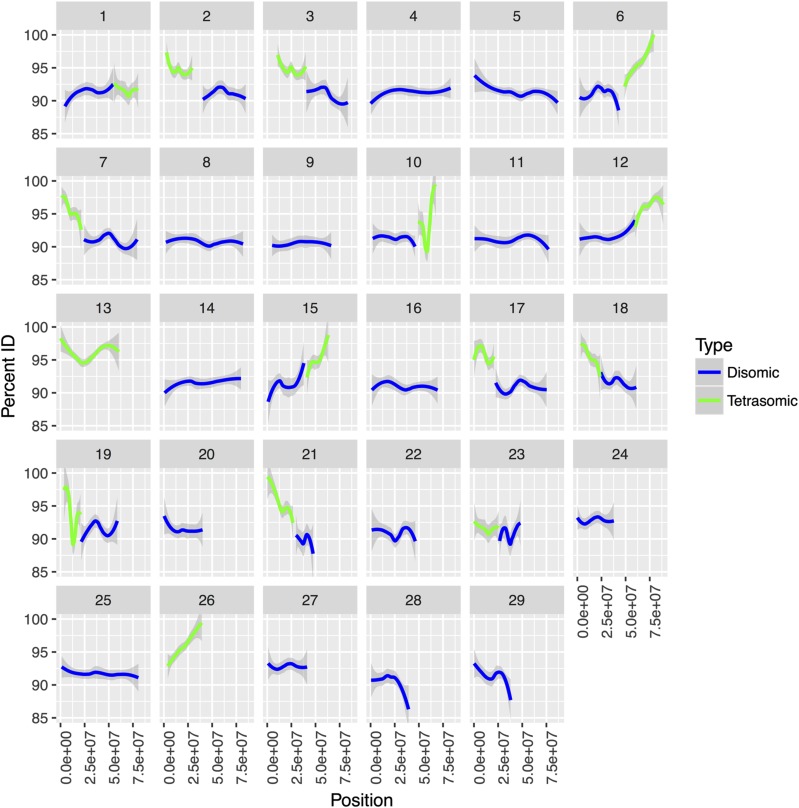
Sequence similarity between ohnologs in different regions of the rainbow trout genome. Disomic inherited regions of the genome are represented in blue and tetrasomic regions are represented in green. The *x* - axis is the position on each chromosome with percent identity in terms of DNA sequence similarity between ohnolog pairs plotted on the *y* - axis.

Data generated from linkage mapping studies and whole genome sequencing projects suggests that eight pairs of homeologous chromosomes still form tetravalent meioses in *Oncorhynchus*, *Salmo*, and *Salvelinus* (*e.g.*, Berthelot *et al.*, 2014; Kodama *et al.*, 2014; Lien *et al.*, 2011; Sutherland *et al.*, 2016). The analysis presented herein reveals that ohnologs in tetrasomic regions of the *O. mykiss* genome have greater sequence similarity compared to ohnologs in disomically inherited regions, presumably due to recombination between homeologs and a subsequent maintenance of genetic variation and gene function. By contrast, ohnologs in disomic regions are able to acquire mutations and diverge, and therefore have lower levels of sequence similarity. Interestingly, we found evidence that one pair of homeologs in rainbow trout (Omy01q and Omy23) shows reduced sequence similarity, in line with levels observed in disomically inherited ohnologs (see Figures 1, 2, and 4). This suggests that tetravalent meioses occur less frequently between Omy01q and Omy23 than the other seven homeologous pairs in rainbow trout. Similar evidence comes from the Atlantic salmon genome, as the homologous chromosome arms to Omy01q and Omy23 (Ssa18qa and Ssa01qa) also show reduced sequence similarity compared to other tetrasomic regions of the Atlantic salmon genome (Lien *et al.*, 2016). We believe this is due to a reduction in the number of tetravalent meioses between Omy01q and Omy23 and a faster return to a disomic state than the other tetrasomic chromosomal regions. Further genome sequencing studies in *Salvelinus* and other *Oncorhynchus* will help confirm if reduced evidence for tetrasomy exists in the homologs of Omy01q and Omy23.

**Figure 3:**
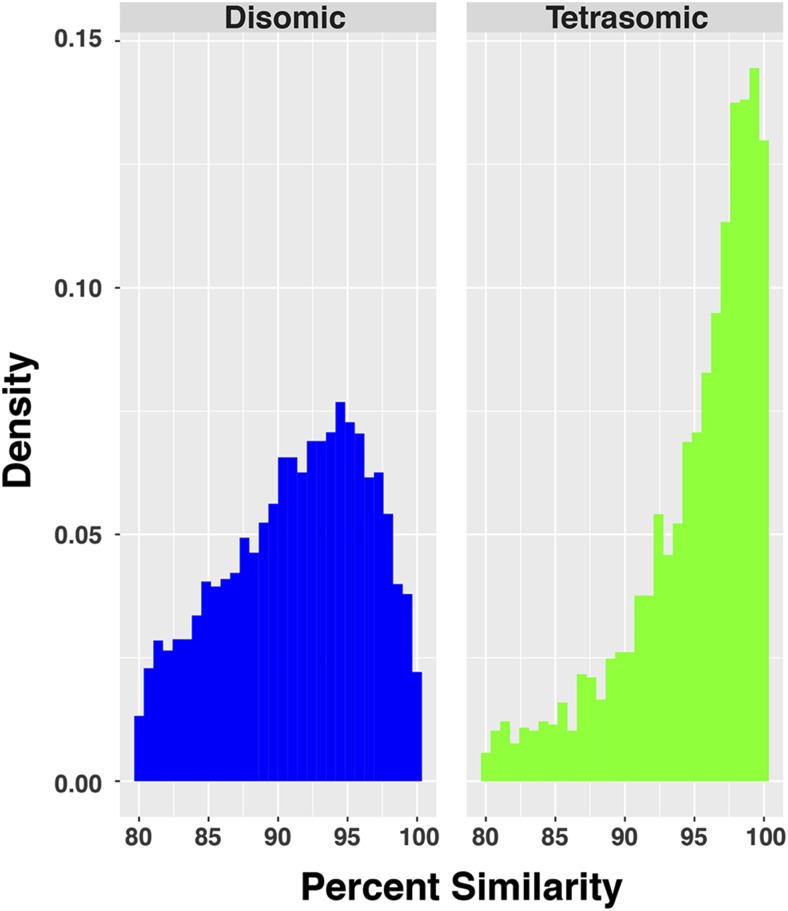
Histograms of the distribution of DNA sequence percent similarity (PID) of ohnolog pairs within disomic (blue) and tetrasomic (green) regions. Mean disomic PID = 91.28, n = 11,402. Mean tetrasomic PID = 94.98, n = 4,556. The means are significantly different (Welch Two Sample t-Test, t = -43.989, df = 9133.3, p-value < 2.2e-16).

**Figure 4:**
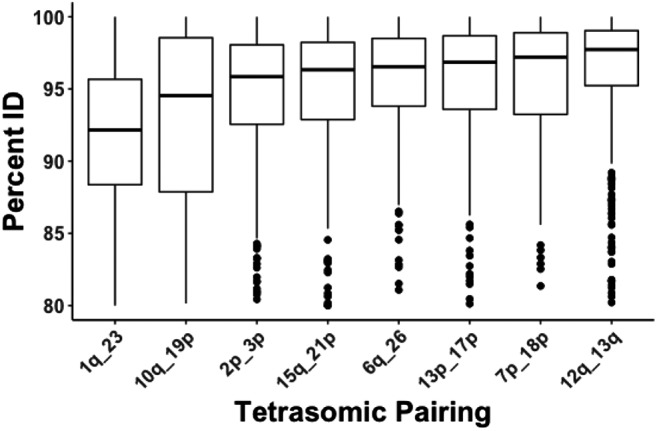
Box plot showing variance in DNA sequence percentage identity between ohnologs located in tetrasomic regions of the genome. Ohnologs are split into eight groups based on their locations in known tetrasomic regions of the rainbow trout genome. Lines represent the median value for, boxes the first and third quartiles and whiskers represent the minimum and maximum value.

**Figure 5:**
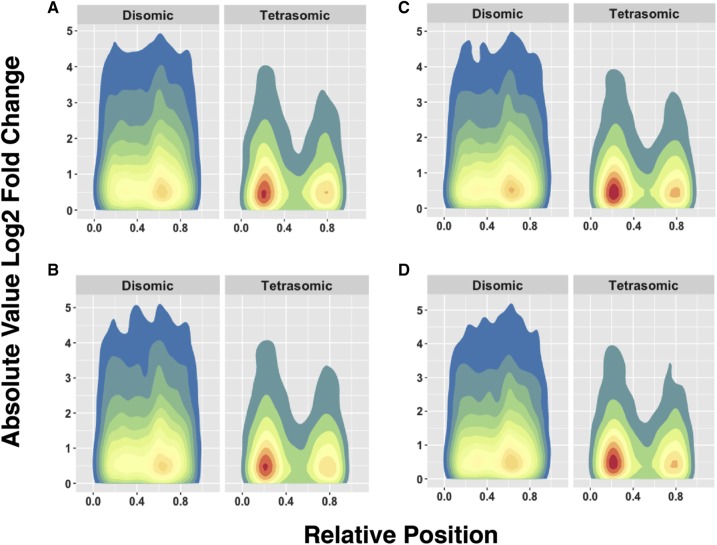
Two dimensional kernel density estimates of the absolute value of log_2_ fold change of ohnolog pairs located in disomic and tetrasomic genomic regions for (A) the female progeny of the A x A cross; (B) the male progeny of the A x A cross; (C) the female progeny of the R x R cross; and, (D) the male progeny of the R x R cross. The plot is color-coded by the density of (*x,y*) observations, where the *x* - axis is relative position along chromosomes, and the *y* - axis is the absolute value of log_2_ fold change between ohnologs. Red indicates a high density of observations, and blue indicates a low density. Tetrasomically inherited ohnologs are overall more similar in expression level than disomically inherited ohnologs and are clustered near the telomeric ends of chromosomes.

### Dangerous duplicates and dosage compensation retain tetrasomic ohnologs

Although gene loss following WGD is the ultimate fate of most ohnologs it is clear that some ohnologs are retained (*e.g.*, Kellis *et al.*, 2004; Wolfe and Shields 1997). Maintaining two functional copies of a gene is thought to require the action of selection to either reduce the accumulation of mutations which leads to either nonfunctionalization or neofunctionalization (Braasch *et al.*, 2018; Sandve *et al.*, 2018), and/or to reduce the expression of both duplicates to match the expression pre-duplication. These two hypotheses both explain how some ohnologs but not others are maintained post duplication. Genes predisposed to haploinsufficiency may be more likely to be retained as functional duplicates to counteract the potential negative effects of mutations in one copy, that is, the dangerous duplicates hypothesis (Singh *et al.* 2012; 2015). However, selection to maintain duplicates and prevent haploinsufficiency has been suggested to be weak, limiting the potential for this explantion for duplicate retention (Pires and Conant; 2016). Nontheless, duplicates involved in immune function, cell cycle regulation, transcriptional control, and cell signaling pathways have been found to be retained during vertebrate evolution (*e.g.*, Freeling and Thomas 2006; Makino and McLysaght 2010). However, enrichment analysis of GO terms presented herein found limited evidence of genes connected to such functions in tetrasomic regions of the genome. Instead, many of the enriched GO terms are connected to functions such as oxidoreductase activity, regulation of hydrogen peroxide, iron binding, and oxygen binding (Table 3). These functions may well be important in the development of life-history variation in salmonids and the smoltification process more broadly, suggesting that that selection can cause the retention of different ohnologs post-duplication depending on the organism-specific factors (*e.g.*, ecological niche).

In addition to trying to understand why some regions of the genome have been maintained as tetrasomic, we also wanted to explore if tetrasomic inheritance modifies the way ohnologs are expressed. The doubling of DNA post whole genome duplication could have a substantial effect on gene expression if, as theorized, duplicates quickly become non-functionalized and silenced. One mechanism that can reduce the effects of dosage imbalance after nonfunctionalization begins is reducing gene expression of both members of an ohnolog pair to equal gene expression pre-duplication (*i.e.*, dosage compensation; Hughes *et al.* 2007). Previous studies in yeast, mice, and *Paramecium* suggest that duplicated genes show lower expression levels than non-duplicated genes regardless of function (*e.g.*, Qian *et al.*, 2010; Aury *et al.* 2006) and that genes involved in protein complexes with multiple subunits should be preferentially retained as duplicates (Roux *et al.*, 2017; Veitia *et al.*, 2008). We found evidence of reduced expression of ohnologs in tetrasomic regions compared to disomic regions in the brain transcriptome of rainbow trout (see Table 2) and evidence of differences in protein-protein interactions between tetrasomic and disomic ohnologs. Veitia *et al.* (2008) suggested that proteins involved in complex interactions (*i.e.*, those with at least two different polypeptides and three subunits) should be more sensitive to dosage imbalance than proteins with more simple interactions. We divided genes into five groups of which one (heteromultimer) were suggested by Roux *et al.* (2017) to be the most sensitive to dosage imbalance. If retention of ohnologs in tetrasomic regions is due to sensitivity of genes to dosage imbalance, then it would be expected that genes involved in heteromultimers should be enriched in tetrasomic regions, which is not what we observed (p – value > 0.05 for each comparison; see Supplemental File S6). This might suggest a lack of support for dosage imbalance as a reason for retention of ohnologs in tetrasomic regions. However, it must be stressed that the number of ohnologs with known protein-protein interactions was small (especially for comparisons to the zebra fish and Atlantic salmon UniProtKB/Swiss-Prot databases) and this lack of significance could be an artifact of small sample size. In addition, we also see a difference in gene function, as previous studies have suggested that many of the ohnologs used to support the dosage-balance hypothesis are involved in essential cellular processes such as metabolism, transcriptional control, and translation. Again, our enrichment analysis does not suggest that genes with these functions are any more likely to be located in tetrasomic regions than disomic regions. Although it is certainly possible that the retention of many ohnologs in tetrasomic regions could be caused by selection impeding neofunctionalization and/or nonfunctionalization because any change would cause alterations in dosage-balance, it is highly likely that different processes are responsible for maintaining both copies of an ohnolog pair in the rainbow trout genome.

Although we demonstrate that gene expression is reduced in ohnologs in tetrasomic regions of the rainbow trout genome, it is important to be mindful of the tissue type investigated. The brain is a key part of the neural system, and previous studies in a range of species (*e.g.*, zebrafish and mice) suggest that genes important in the neural system are a) highly expressed, b) highly conserved, and c) contain a high proportion of ohnologs (Roux *et al.*, 2017). Roux *et al.* (2017) consistently found that genes expressed in neural tissue are more likely to be retained in duplicates after WGD. Although few studies have investigated how tissue specific expression influences the process of ohnolog retention, many studies have found genes annotated with functions connected to neurodevelopment, cell signaling, and behavior to be more likely to be maintained as ohnologs than genes with other functions (*e.g.*, Brunet *et al.*, 2006; Howe *et al.*, 2013; Putnam *et al.*, 2008). Although it is impossible to make similar inferences between tissues with the data herein (especially as brain tissue is complex), future studies that aim to investigate expression patterns of ohnologs across different salmonid species, different tissues, and different time points would be enlightening. It is possible, that the patterns of expression discussed herein are time, tissue, population, or species specific, although this seems unlikely both due to previous studies that have documented a reduction in expression post duplication, and the conservation of homologous tetrasomic regions in *Salmo*, *Salvelinus*, and *Oncorhynchus*. Ultimately, gene expression reduction could be a mechanism that maintains duplicates, preventing them from returning to a diploid state. The enrichment of ohnologs in tetrasomic regions compared to disomic regions may suggest that genes located on these regions are essential for salmonid function and development.

### The salmonid-specific WGD and the development of anadromy

The anadromous life cycles of many salmonids has long inspired debate about the potential link between whole-genome duplication and anadromy (Alexandrou *et al.*, 2013; McDowall 2002). The propensity to migrate *vs.* maintain residency in rainbow trout is associated with genetic polymorphisms, gene expression differences, and differential methylation patterns (Baerwald *et al.*, 2015; Hale *et al.*, 2016, 2013; Hecht *et al.*, 2015, 2012; McKinney *et al.*, 2015; Pearse *et al.*, 2014, 2018). Our GO analysis suggests that genes connected to several metabolic processes, such as oxygen binding and oxidoreductase activity, are located in tetrasomic regions of the rainbow trout genome. Such functions have an obvious connection to life history variation and the development of anadromy. Moreover, Robertson *et al.* (2017) suggests that several ohnolog pairs involved in hormonal regulation and ion transport including insulin growth factor 1 and thyroid hormone receptor alpha (*i.e.*, processes strongly linked with smoltification) are located in tetrasomic regions of the Atlantic salmon genome. However, many other genes known to be involved in anadromy, including the major chromosomal rearrangement on chromosome Omy05 (Pearse *et al.* 2018), are not located in tetrasomic regions (*e.g.*, many hormone receptors, genes involved in maintenance of ionic balance, and growth genes). Therefore, any link between anadromy and the salmonid WGD is circumspect, as it is clear that this complex phenotype is caused by many molecular pathways located in different regions of the salmonid genome. Moreover, multiple studies suggest that, at least in rainbow trout, the molecular control of anadromy varies between different populations, suggesting plasticity in the development of different life history strategies making it unlikely that the WGD is, at least currently, a major factor in life history development (*e.g.*, Hale *et al.*, 2013; Hecht *et al.*, 2013, 2012; Nichols *et al.*, 2008).

### Conclusions

Tetrasomic regions of the rainbow trout genome contain a higher density of ohnologs, are more similar in their sequence identity, have lower overall expression, and are expressed more similarly than ohnologs in disomic regions. The conservation of a large portion of ohnologs post-WGD in rainbow trout may be because modification of these ohnologs results in negative effects on the organism. Understanding the effects on genome organization of WGD is hampered by difficulties in identifying ancient autopolyploids. The identification and study of additional teleost polyploid lineages could lead to a more thorough understanding on the fates of genes post-WGD. Why some genes are maintained as duplicates post-WGD, whereas others undergo neofunctionalization are important questions to pursue in the field of polyploidy research. Ultimately, our understanding of such processes is strongly biased toward plants and mammals. Additional research in more lineages of fishes may prove insightful in our understanding of the consequences of WGD and the identification of general outcomes from lineage-specific outcomes.
